# Phycocyanin Improves Reproductive Ability in Obese Female Mice by Restoring Ovary and Oocyte Quality

**DOI:** 10.3389/fcell.2020.595373

**Published:** 2020-11-13

**Authors:** Xin Wen, Zhe Han, Shu-Jun Liu, Xin Hao, Xiao-Jie Zhang, Xing-Yue Wang, Cheng-Jie Zhou, Yu-Zhen Ma, Cheng-Guang Liang

**Affiliations:** ^1^State Key Laboratory of Reproductive Regulation and Breeding of Grassland Livestock, School of Life Sciences, Inner Mongolia University, Hohhot, China; ^2^Inner Mongolia People’s Hospital, Hohhot, China

**Keywords:** phycocyanin, obesity, ovary, oocyte, offspring, oxidative stress

## Abstract

Reproductive dysfunction associated with obesity is increasing among women of childbearing age. Emerging evidence indicates that maternal obesity impairs embryo development and offspring health, and these defects are linked to oxidative stress in the ovary and in oocytes. Phycocyanin (PC) is a biliprotein from *Spirulina platensis* that possesses antioxidant, anti-inflammatory, and radical-scavenging properties. Our previous studies have shown that PC can reduce reactive oxygen species (ROS) accumulation in oocytes in D-gal-induced aging mice. Here, at the Institute of Cancer Research (ICR) mice fed a high-fat diet (HFD) to model obesity were used to test the effect of PC on reversing the fertility decline caused by obesity. We observed a significant increase in litter size and offspring survival rates after PC administration to obese mice. Further, we found that PC not only ameliorated the level of ovarian antioxidant enzymes, but also reduced the occurrence of follicular atresia in obese female mice. In addition, the abnormal morphology of the spindle-chromosome complex (SCC), and the abnormal mitochondrial distribution pattern in oocytes both recovered. The obesity-related accumulation of ROS, increased number of early apoptotic cells, and the abnormal expression of H3K9me3 in oocytes were all partially reversed after PC administration. In summary, this is the first demonstration that PC can improve fertility by partially increasing ovarian and oocyte quality in obese female mice and provides a new strategy for clinically treating obesity-related infertility in females.

## Introduction

The proportion of obese people has increased significantly as the general quality of life has improved (Darbre, 2017), and maternal obesity has been reported to cause a variety of diseases (Rogero and Calder, 2018). For example, obesity can reduce ovarian and oocyte quality, increase the level of oxidative stress in them (Igosheva et al., 2010; Luzzo et al., 2012; Grindler and Moley, 2013), and ultimately damage female fertility (Jungheim and Moley, 2010; Silvestris et al., 2018). Evidence also indicates that obese females have a lower probability for conception and a higher risk for miscarriages, preeclampsia, and congenital defects in their offspring (Machtinger et al., 2012). In addition, maternal obesity has been associated with spindle defects and chromosome misalignment during meiotic oocyte maturation (Jungheim et al., 2010; Luzzo et al., 2012), and obesity-related maternal metabolic syndrome induced structural, spatial, and metabolic alterations in oocyte mitochondria (Reynolds et al., 2015; Saben et al., 2016). Reactive oxygen species, (ROS), a by-product of oxidative phosphorylation, are simultaneously produced in mitochondria (Balaban et al., 2005), and their levels were dramatically elevated in obese female oocytes (Zhang et al., 2015). Previous reports have demonstrated that ROS accumulation in cells leads to cytoskeleton abnormalities (Yancey et al., 2015), antioxidant system dysfunction (Janda et al., 2016), abnormal distribution of mitochondria (Yu et al., 2016), and cell apoptosis (Liu et al., 2016). Therefore, obesity is harmful to female reproductive capability (Catalano and Ehrenberg, 2006).

A recent prospective study has suggested that specific dietary ingredients may improve obesity-induced reproductive impairment (Han et al., 2017). Phycocyanin (PC) is a major biliprotein extracted from *Spirulina platensis*, and is mostly found in red algae, cyanobacteria, and cryptophaga (Benedetti et al., 2004). As an antioxidant, PC was found to inhibit cell aging and to protect mitochondrial function in many cell types (Fernández-Rojas et al., 2014). Recently, we found that continuous intragastric administration of PC significantly reduced the accumulation of ROS inside oocytes of D-galactose-induced aging mice and improved their oocyte quality, increasing female fertility (Li et al., 2016). Moreover, increasing evidence indicates that PC promotes cell activity, eliminates free radicals, and improves organ function (Li et al., 2013; Hao et al., 2018), and has become widely used as a natural substance with antioxidant, neuro-protective, anti-inflammatory, and oxygen free-radical scavenging properties (Romay et al., 2003; Gupta et al., 2011; Fernández-Rojas et al., 2014).

The negative impact of obesity on oocyte quality is well known (Sohrabi et al., 2015), and the fact that PC improves oocyte quality has also been well-documented (Li et al., 2016), but the effects of PC on ovaries and oocytes damaged by obesity remains unclear. Therefore, the purpose of the present research was to determine whether PC could alleviate the negative impact of obesity on ovary, oocyte quality and fertility. We found that PC did maintain ovary/oocyte quality and fertility in obese mice, and these results may be important for treating obesity-related subfertility in humans.

## Materials and Methods

### Experimental Design, Mouse Feeding, Mating, and Offspring Assessments

**FIGURE 1:**
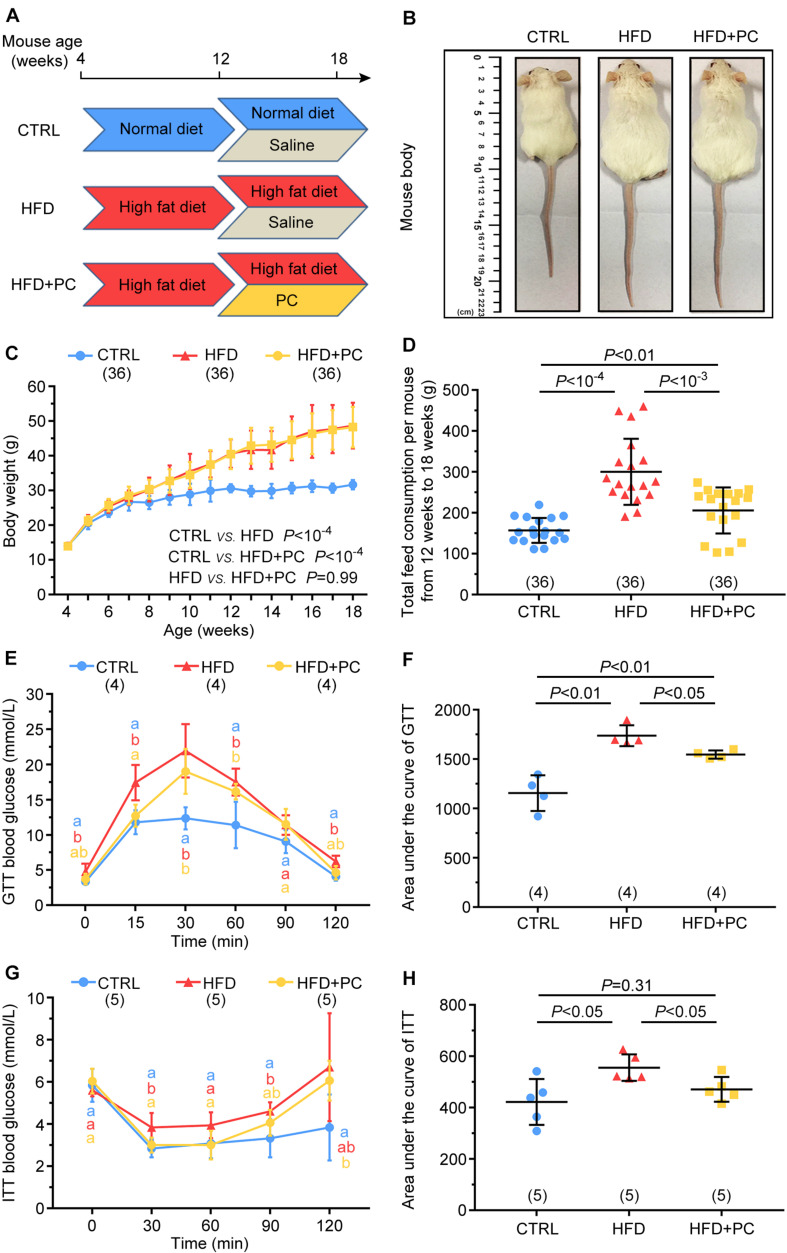
PC improved glucose tolerance and insulin resistance in obese female mice. **(A)** Experimental design. The mice in the CTRL group were fed a normal diet (blue) and had intragastric administrations of saline (gray). Mice in the HFD group were fed a high-fat diet (red) and had intragastric administrations of saline (gray). The mice in the HFD + PC group were fed a high-fat diet (red) and had intragastric administrations of PC (yellow). **(B)** Image of mice at the age of 18 weeks. **(C)** Weight changes in mice (4–18 weeks of age). **(D)** Average food consumption (12–18 weeks of age). **(E)** Blood glucose changes in GTT. **(F)** GTT AUC values. **(G)** Blood glucose changes in ITT. **(H)** ITT AUC values. Data are expressed as mean ± SD and were compared by one-way ANOVA and Newman–Keuls *post hoc* tests. In **(E,G)**, the different letters at the same time point near the error bars indicate statistical significance (*p* < 0.05). The number of mice in each group is shown in parentheses.

All research procedures conformed to the National Research Council Guide for the Care and Use of Laboratory Animals (Garber et al., 2011), and were approved by the Institutional Animal Care and Use Committee at the Inner Mongolia University (Approval number: SYXK 2014-0002). ICR mice were purchased from the Research Centre for Laboratory Animal Science of Inner Mongolia University, and were reared in a special pathogen free animal facility in the Research Centre for Laboratory Animal Science of Inner Mongolia University under the conditions of a 12:12-h light:dark cycle. Throughout the experiments, mice were free to get food and water. At the age of 4 weeks, female mice were randomly divided into three groups and treated as follows. In the control group (CTRL), mice were continuously fed a normal diet (H10010, HFK Bioscience Co., Ltd., Beijing, China) for 14 weeks. During the last 6 weeks, 0.4 ml of normal saline was administered intragastrically each day. In the high-fat diet group (HFD), mice were continuously fed a high-fat diet (D12492, Research Diets Inc., New Brunswick, NJ, United States) for 14 weeks. During the last 6 weeks, 0.4 ml saline was administered intragastrically each day. In the high-fat diet + phycocyanin group (HFD + PC), mice were continuously fed a high-fat diet for 14 weeks. During the last 6 weeks, PC (Zhejiang Binmei Biotechnology Co., Ltd, Taizhou, Zhejiang, China) at a dose of 500 mg/kg/day (dissolved in ultrapure water at a concentration of 50 mg/ml) was administered intragastrically each day. A schematic of the experimental design is shown in Figure 1A. Food consumption, and mouse body weights were recorded weekly.

If body weight increased more than 20% compared to controls, mice were defined as obese (Lee et al., 2020). In terms of the PC dosage, its intragastric administration at 500 mg/kg/day improved the reproductive abilities of aging female mice (Li et al., 2016), so we used this dose in the current study.

A single ICR male mouse at 12 weeks of age (with proven fertility) was caged overnight with a single female mouse for mating. Vaginal plugs in the female mice were detected in the morning of the next day. The numbers, weights, and survival rate of the offspring were recorded weekly from delivery to the age of 8 weeks. The sex ratio (male/female) of the pups was determined at 3 weeks of age.

### Glucose Tolerance Tests (GTTs) and Insulin-Tolerance Tests (ITTs)

Mice were injected intraperitoneally with glucose (2 g/kg body weight) after 12 h of fasting before GTT analysis. Mice were injected intraperitoneally with insulin (0.75 IU/kg body weight) after 4 h of fasting before ITT analysis. Blood was gathered from the tail vein at the appropriate time point. GTT and ITT levels were measured with the blood glucose meter (Roche, Basel, Switzerland, Germany). GraphPad Prism 7 software was used to calculate area under the curve (AUC) values for both GTT and ITT. *Glucose tolerance* refers to a reduction in the body’s ability to regulate blood glucose levels (González-Grajales et al., 2018), and *insulin resistance* refers to a reduction in the response of a peripheral target tissue to a physiological concentration of insulin (Hotamisligil et al., 1993).

### Ovary Histology and Follicle Counting

Mouse ovaries were harvested and fixed in 4% paraformaldehyde (Electron Microscopy Sciences, Hatfield, PA, United States) overnight at 4°C, followed by dehydration with ethanol. Paraffin-embedded ovaries were sectioned serially (5 μm thickness) and every fifth section was mounted on slides for hematoxylin and eosin (H&E) staining (Nanjing Jiancheng Bioengineering Institute, Nanjing, Jiangsu, China) and neutral resin fixation, then examined by optical microscopy (Nikon ECLIPSE Ci, Tokyo, Japan). For each section, only follicles in which the oocyte nucleus could be clearly visualized were scored. Follicle classifications were made as previously described (Wang et al., 2014), and the numbers of follicles at the different developmental stages were counted.

### Quantitative Real-Time Polymerase Chain Reaction (RT-PCR) and Biochemistry Assays

For quantitative RT-PCR, total RNA was extracted from ovaries using the TaKaRa MiniBEST Universal RNA Extraction Kit (TaKaRa, Dalian, Liaoning, China) according to the manufacturer’s instructions. cDNA was synthesized using the PrimeScript RT reagent Kit (TaKaRa) following the manufacturer’s instructions. The primer sequences were as follows: *BMP4* (Forward: 5′-TCCTGGTAACCGAATGCTGAT-3′; Reverse: 5′-GCTGCTGAGGTTGAAGAGGAA-3′), *GDF9* (Forward: 5′-AATACCGTCCGGCTCTTCAG-3′; Reverse: 5′-G GTTAAACAGCAGGTCCACCAT-3′), *LHX8* (Forward: 5′-CAG TTCGCTCAGGACAACAA-3′; Reverse: 5′-CCTGCAGTTC TGAAACCACA-3′), *GAPDH* (Forward: 5′-CGGCCGCATC TTCTTGTG-3′; Reverse: 5′-CCGACCTTCACCATTTTGTC TAC-3′). RT-PCR was performed with the SYBR Green kit (TaKaRa). The comparative Ct method was used for data analysis, and GAPDH was used as an internal control.

The levels of oestradiol and follicle stimulating hormone (FSH) in serum were measured using an ELISA kit (CUSABIO, Wuhan, Hubei, China). Ovarian catalase (CAT), glutathione peroxidase (GSH-Px), superoxide dismutase (SOD) activities, and malondialdehyde (MDA) content were measured using their corresponding kits (Nanjing Jiancheng Bioengineering Institute). All detection procedures were carried out according to the manufacturers’ instructions.

### Oocyte Collection, Maturation, Fertilization, and Embryo Culture

The oocytes in GV phase were harvested from female mice 48 h after injection of 5 IU pregnant mare serum gonadotropin (PMSG, Ningbo Sansheng Pharmaceutical Co., Ltd., Ningbo, Zhejiang, China) by puncturing the ovary follicles. Cumulus-oocyte complexes (COCs) were gently transferred into M2 medium to remove cumulus cells. Oocytes were washed and cultured in Chatot-Ziomek-Bavister (CZB) media in a humidified atmosphere of 5% CO_2_ at 37°C for maturation. Germinal vesicle breakdown (GVBD) and the first polar body (PB1) were observed 2.5 h and 14 h later, respectively.

For metaphase II (MII) oocytes matured *in vivo*, mice received an intraperitoneal injection of 5 IU PMSG followed by administration of 5 IU human chorionic gonadotropin (hCG, Ningbo Sansheng Pharmaceutical Co., Ltd.) 48 h later. The COCs were released from the oviduct ampullae 14 h after hCG injection. Denuded MII oocytes were obtained by removing the cumulus mass in M2 medium containing 0.3 mg/ml hyaluronidase. PB1 extrusions and oocyte fragmentations were examined.

Oocytes fertilization and early embryonic development were evaluated as previously described (Zhou et al., 2016). Zygotes that developed to 2-cell stage embryos were classified as successfully fertilized oocytes. Embryos were evaluated at 48 h, 72 h, and 96 h after fertilization, and the percentages of embryos at the 4-cell stage, morula stage and blastocyst stage were calculated, respectively.

### Immunofluorescence and Confocal Microscopy

Immunofluorescence detecting was performed as previously described (Li et al., 2016). For primary antibodies, we used mouse anti-beta-tubulin (1:1000, Abcam, Cambridge, United Kingdom) and anti-H3K9me3 (1:500, Abcam). For secondary antibodies, we used DyLight 549-conjugated donkey anti-mouse (1:100, Jackson Immuno Research Laboratories, West Grove, PA, United States). DNA was stained with 4′,6-diamidino-2-phenylindole (DAPI, 5 μg/ml, Roche, Mannheim, Germany) for 10 min. After washing, samples were mounted onto glass slides and examined with a confocal laser-scanning microscope (Nikon A1R).

### Determining the Distribution of Mitochondria

For determining mitochondrial distribution, MII oocytes were fixed in 4% paraformaldehyde for 30 min in a humidified chamber, and stained with 25 μM MitoTracker Green FM (Molecular Probes, Eugene, OR, United States) for 30 min and 50 ng/ml DAPI for 10 min in the dark. After staining, the samples were mounted onto microscope slides and examined using confocal microscopy as above. The distribution patterns of mitochondria in oocytes were divided into two types: evenly distributed or aggregated. Normally, mitochondria are evenly distributed in MII oocytes (Jia et al., 2018), so *mitochondria-even* denoted this evenly distributed pattern, whereas *mitochondria-aggregation* denoted a distribution pattern where mitochondria were clumped/aggregated.

### Mitochondrial Membrane Potential (ΔΨm) Measurements

The ΔΨm was assessed using the mitochondrial inner membrane potential dye JC-10 (Beyotime, Hangzhou, Zhejiang, China), according to the manufacturer’s instructions. Briefly, oocytes were placed in a working solution with a final concentration of 10 μM JC-10, and cultured in darkness at 37°C, 5% CO_2_ for 20 min. Samples were assessed using confocal microscopy as above. The mitochondrial membrane potentials were calculated using the ratio of red to green fluorescence intensities in the oocytes.

### ROS Assay

To evaluate ROS production inside oocytes, oocytes were incubated in CZB media containing 10 μM DCFH-DA (Nanjing Jiancheng Bioengineering Institute) for 30 min at 37°C in a 5% CO_2_ incubator. Oocytes (10–15) were then transferred to a cell-imaging dish, and the fluorescence intensity of each oocyte was measured using confocal microscopy as above.

### Annexin-V Staining

Oocyte apoptosis was evaluated using the Annexin-V-FITC Apoptosis Kit (Vazyme, Nanjing, Jiangsu, China). Oocytes were stained with 195 μl binding buffer containing 5 μl Annexin-V-FITC for 30 min in the dark. After washing three times, fluorescence signals were detected using confocal microscopy as above, with oocyte-membrane fluorescence as an indicator of early apoptosis.

### Statistical Analysis

Results are shown as means ± standard deviations (SD) representing more than three replicate experiments. Statistical comparisons were made using an analysis of variance (ANOVA), and differences among the three groups were assessed using Newman–Keuls multiple-comparison *post hoc* tests. For the female mouse vaginal plug rate, offspring gender rate, and mitochondrial distributions, chi-squared tests in Microsoft Excel software (Microsoft Corporation, Redmond, WA, United States) were used. The offspring survival rate was analyzed using the log-rank test (Mantel-Cox). All other analyses were performed using GraphPad Prism 7.0 statistical software (GraphPad Software Inc., La Jolla, CA, United States). A *p* value < 0.05 was considered statistically significant.

## Results

### PC Improved Glucose Tolerance and Insulin Resistance in Obese Female Mice

The weights of mice in each group were measured before feeding to ensure that all mice were comparable. With increasing age and food intake, the weight gains for the HFD and HFD + PC groups were significantly higher compared to the CTRL group (*p* < 10^–4^). Intragastric administration of PC did not affect the body weights of HFD mice (Figures 1B,C). However, it did reduce food consumption in HFD mice between 12 and 18 weeks (Figure 1D).

The GTT analysis showed that glucose concentrations in the HFD group were higher than those in the CTRL and HFD + PC groups at 15 and 30 min (*p* < 0.05) (Figure 1E). Further AUC analyses confirmed these results (Figure 1F). Similarly, the ITT blood test results (Figure 1G) and the corresponding AUC values (Figure 1H) also showed a significant increase in insulin resistance in the HFD group, indicating intragastric administration of PC improved the glucose metabolism and insulin response in obese female mice.

### PC Increased Litter Size and Offspring Viability in Obese Mice

**FIGURE 2:**
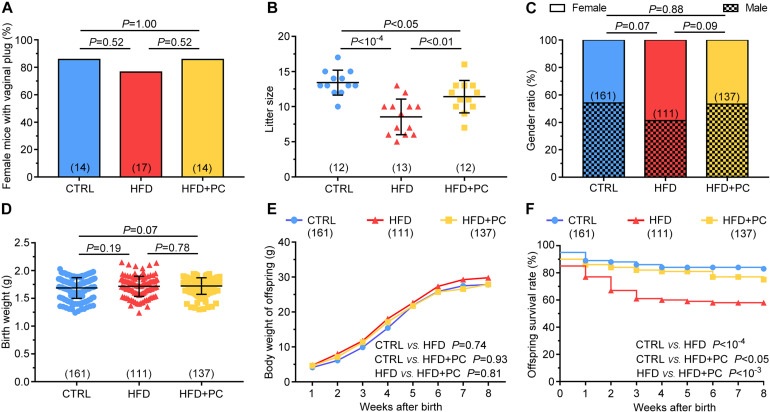
PC increased litter sizes and offspring viability in obese female mice. **(A)** A comparison of mating rates in female mice. **(B)** Offspring litter sizes. **(C)** The gender ratios for offspring. **(D)** Offspring birth weights. **(E)** Body weights for offspring (1–8 weeks of age). **(F)** Offspring survival rates (0–8 weeks of age). Data are expressed as mean ± SD and were compared by one-way ANOVA and Newman–Keuls *post hoc* tests for **(B,C)**, chi-squared tests for **(A,E)**, log-rank tests (Mantel-Cox) for **(D,F)**. The number of mice in each group is shown in parentheses.

Phycocyanin’s improvement effect on obese female mice fertility was also assessed by evaluating the offspring. The mating probabilities of females (indicated by vaginal plugs) in the CTRL, HFD, and HFD + PC groups were comparable (*p* > 0.05), illustrating that a HFD or PC gavage did not affect female mating ability (Figure 2A). However, litter size in the HFD group was significantly lower when compared to the CTRL group (*p* < 10^–4^). The litter size reduction after HFD treatment was partially reversed by PC administration (*p* < 0.01) but was still lower compared to controls (*p* < 0.05) (Figure 2B). In terms of the gender ratios, birth weights, and body weights from 1 to 8 weeks after birth, there were no significant differences among the three groups (*p* > 0.05) (Figures 2C–E). To our surprise, there was a significant decrease in offspring survival (1–8 weeks after birth) found in the HFD group. Only 58% of the offspring survived after 8 weeks in the HFD group, compared to the CTRL (83%) or the HFD + PC group (75%) (Figure 2F). These results indicate that obesity in females has a negative effect on both litter size and offspring survival that can be partially reversed by PC administration.

### PC Prevented Ovarian Follicular Atresia, and Improved FSH Levels and Antioxidant Enzyme Activity in Obese Mice

**FIGURE 3:**
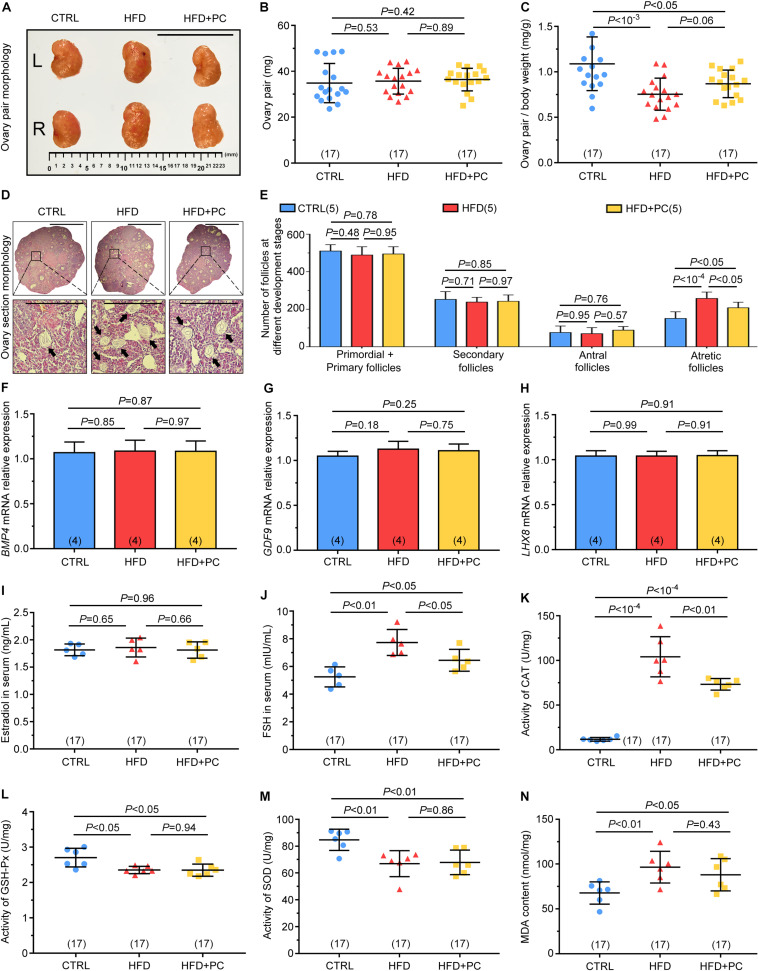
PC promoted follicular development and reduced CAT and FSH levels in obese mice. **(A)** Macroscopic representations of the ovaries, scale bar = 10 μm. **(B)** Ovary-pair weights. **(C)** Ovary-pair ratio coefficients. **(D)** Typical histological image of H&E-stained follicles, scale bar = 50 μm, with black arrows indicating atretic follicles. **(E)** The statistics for follicles at different developmental stages in the ovaries. **(F)** Relative expressions of *BMP4* mRNA. **(G)** Relative expressions of *GDF9* mRNA. **(H)** Relative expressions of *LHX8* mRNA. **(I)** Estradiol levels. **(J)** FSH levels. **(K)** CAT activities. **(L)** GSH-Px activities. **(M)** SOD activities. **(N)** MDA contents. Data are expressed as mean ± SD and were compared by one-way ANOVA and Newman–Keuls *post hoc* tests. The number of ovary pairs in each group is shown in parentheses.

To understand the ability of PC to reverse the HFD effect on female mouse reproductive ability, both ovaries and follicles were assessed. There were no significant gross morphological differences among the three groups of ovary pairs (Figure 3A), and ovary-pair wet weights were comparable among the three groups (Figure 3B). However, the ovary-ratio coefficient in the HFD group was much lower than that for the CTRL group due to HFD-increased body weights (*p* < 10^–3^), and PC administration did not reverse these changes in the ovary-ratio coefficient in HFD mice (*p* < 0.05) (Figure 3C). The number of ovary follicles at different developmental stages were also assessed using histology (Figure 3D). There were no significant differences among the three groups in terms of the number of primordial and primary follicles, secondary follicles, or antral follicles (*p* > 0.05). However, the number of atretic follicles in the HFD group was significantly higher than the number in the CTRL group (*p* < 10^–4^). Although PC administration reduced the number of atretic follicles in the HFD mice group (*p* < 0.05), this number was still higher compared to that in the CTRL group (*p* < 0.05) (Figure 3E). These results indicate that PC had the ability to prevent ovarian follicular atresia in obese mice.

Subsequently, we also examined the mRNA expression levels of *BMP4*, *GDF9*, and *LHX8* related to ovarian follicular development. No significant differences were found among the three groups (*p* > 0.05) (Figures 3F–H).

Obesity can disrupt homeostasis and cause hormone disorders. To study the effect of PC on hormones in HFD mice, we assessed the levels of oestradiol and FSH in mice serum. No statistical differences were found in oestradiol levels among the three groups (*p* > 0.05) (Figure 3I). However, FSH levels in the HFD group were significantly higher than those in the CTRL group (*p* < 0.01). After intragastric administration of PC to HFD mice, FSH levels were significantly reduced (*p* < 0.05), but still higher than those in the CTRL group (*p* < 0.05) (Figure 3J).

Next, we measured ovarian activity and content of antioxidant enzymes. Significantly, CAT activity in the HFD group was higher than that in the CTRL group (*p* < 10^–4^), and this increase was partially reversed by PC treatment (*p* < 0.01) (Figure 3K). In the HFD group, the activities of GSH-Px and SOD were decreased compared with their activities in the CTRL group, and this decrease was not reversed after PC administration (Figures 3L,M). Conversely, MDA content in the HFD group was higher compared to the CTRL group (*p* < 0.01), and this abnormality was not reversed by PC treatment (Figure 3N).

### PC Improved Oocyte Quality in Obese Mice

**FIGURE 4:**
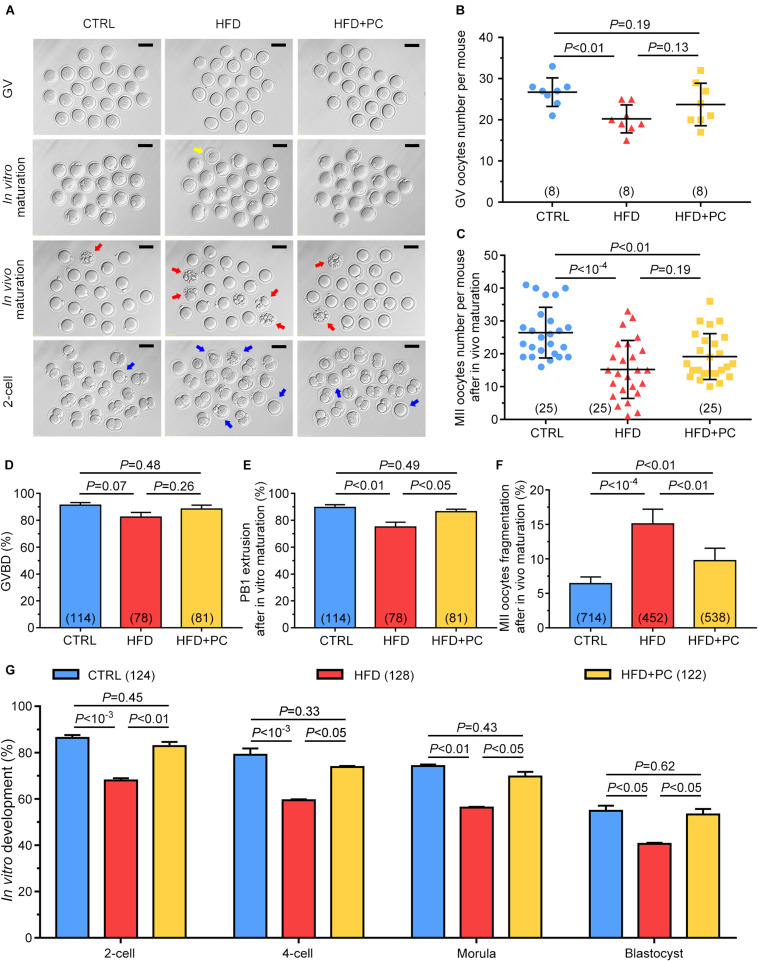
PC rescued oocyte quality in obese mice. **(A)** Representative morphological images of GV, *in vivo* or *in vitro* matured oocytes, and 2-cell stage embryos. The yellow arrow indicates an oocyte with degenerated cytoplasm, red arrows indicate fragmented oocytes, and blue arrows indicate oocytes with failed fertilization or cleavage. Scale bar = 100 μm. **(B)** Number of GV oocytes per mouse. **(C)** Number of MII oocytes per mice after *in vivo* maturation. **(D)** The percentage of oocytes that underwent GVBD. **(E)** The percentage of *in vitro* cultured GV oocytes that underwent PB1 extrusion. **(F)** The percentage of oocytes with fragmentation morphologies. **(G)** The percentage development to 2-cell, 4-cell, morula, and blastocyst embryos. Data are expressed as mean ± SD and were compared by one-way ANOVA and Newman–Keuls *post hoc* tests. The number of mice assessed in each group is indicated in parentheses for **(B,C)**. The number of oocytes or embryos assessed in each group is indicated in parentheses for **(D–G)**.

As litter size was observed to be increased after PC administration in HFD mice, we wanted to know if this increase was due to an improvement in oocyte quality. Oocytes or 2-cell-stage embryos from both *in vivo* and *in vitro* culture models showed impaired developmental potential in the HFD group, indicated by enlarged perivitelline spaces, and fragmented or dark cytoplasm. In contrast, after PC treatment, the majority of oocytes and embryos exhibited normal morphologies comparable to the CTRL group (Figure 4A). After gonadotropin administration, the numbers of GV or MII oocytes superovulated from each mouse were counted. The number of GV oocytes collected from the HFD group was much lower than the CTRL group number (*p* < 0.01). Similarly, the number of MII oocytes from the HFD group was also much lower than the CTRL group number (*p* < 10^–4^). Although the numbers of both GV and MII oocytes increased after PC administration, there were no statistical differences when compared with the HFD group (*p* > 0.05) (Figures 4B,C). For *in vitro* maturation, neither HFD nor PC treatment influenced oocyte meiotic resumption, which was indicated by the comparable occurrence of GVBD (Figure 4D). However, PC rescued impaired-oocyte nuclear maturation potential in HFD mice, indicated by the increased percentage of PB1 extrusions (*p* > 0.05) (Figure 4E). In addition, a high frequency of fragmentation in *in vivo*-matured oocytes was observed in the HFD group (*p* < 10^–4^). PC treatment partially reversed this fragmentation rate (*p* < 0.01), but it was still higher than the CTRL group rate (*p* < 0.01) (Figure 4F). Moreover, our results show that the early development of embryos generated from IVF was affected both by HFD and PC. The embryo development rates for the 2-cell, 4-cell, morula and blastocyst stages in the HFD group were lower than those in the CTRL group (*p* < 0.05). After PC treatment, the developmental potentials for each stage increased significantly (*p* < 0.05) (Figure 4G). Overall, although PC did not increase the number of fully grown oocytes in obese female mice, it significantly improved oocyte quality and embryo development potential.

### PC Reversed Spindle-Chromosome Complex (SCC) Malformations in Obese Mice

**FIGURE 5:**
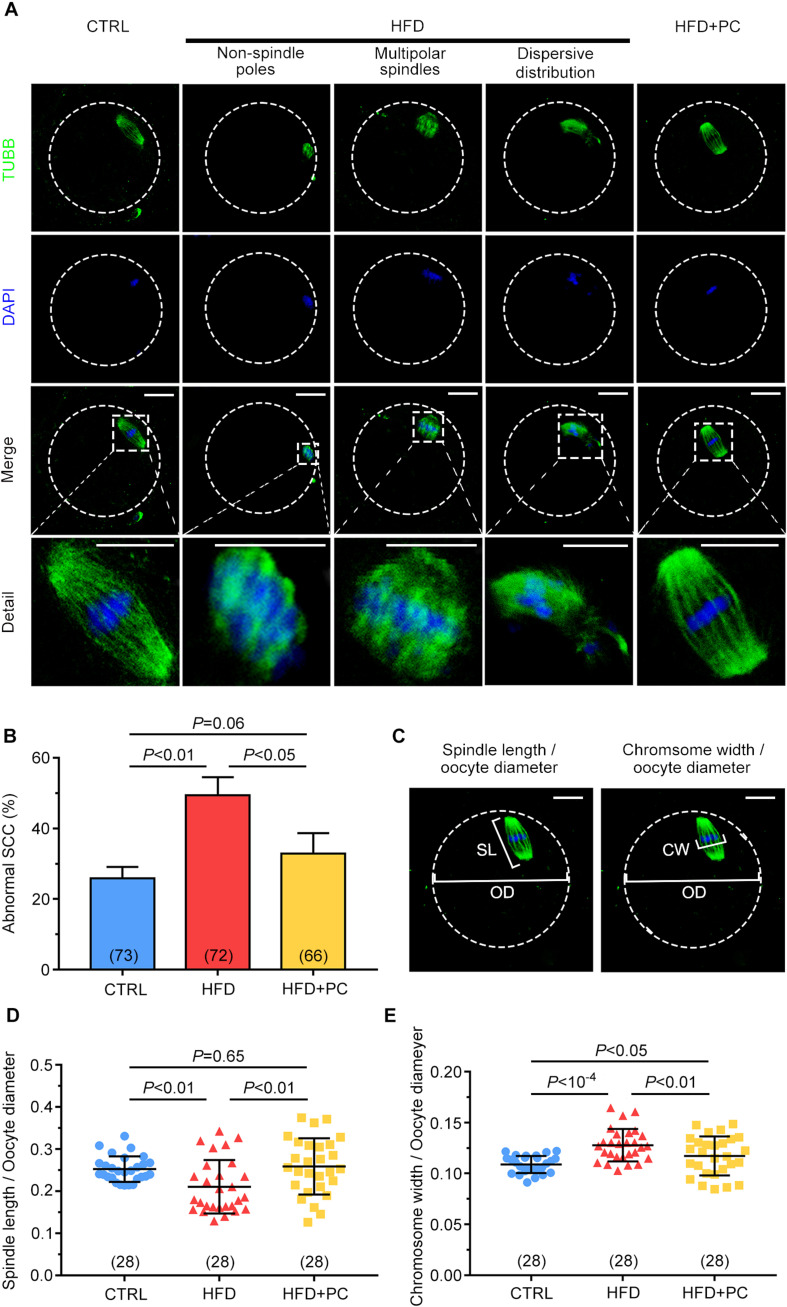
Spindle-chromosome complex (SCC) malformations were reversed after PC administration in obese mice. **(A)** Representative images of SCCs in MII oocytes. Oocytes in the CTRL group exhibited normal spindles and chromosome condensation (left column). Oocytes in the HFD groups displayed abnormal SCCs (three middle columns). Normal oocyte spindle and chromosome condensation in the HFD + PC group (right column). The circular white frame indicates the oocyte edge and the square white square indicates the region shown in detail. Green, tubulin; blue, DNA. Scale bar = 20 μm. **(B)** The ratios of SCC abnormalities in the CTRL, HFD, and HFD + PC groups. **(C)** Schematic representation of spindle length (SL)/oocyte diameter (OD) **(left panel)** and chromosome width (CW)/oocyte diameter (OD) **(right panel)**. Green, tubulin; blue, DNA. The circular white frame indicates the oocyte edge. Scale bar = 20 μm. **(D)** Spindle length/oocyte diameter ratios in MII oocytes. **(E)** Chromosome width/oocyte diameter ratios in MII oocytes. Data are expressed as mean ± SD and were compared by one-way ANOVA and Newman–Keuls *post hoc* tests. The number of oocytes assessed in each group is shown in parentheses.

Staining of beta-tubulin and DNA was carried out to assess the effects of obesity on SCC morphology in MII oocytes. Fusiform-shaped spindles with well-organized chromosomes in metaphase were observed in the majority of oocytes in the CTRL group. In contrast, abnormal chromosome condensation and spindle assembly defects were observed in the HFD groups. These defects included non-spindled poles, multipolar spindles, and dispersed distributions. It is worth noting that these defects were partially reversed in oocytes after PC administration (Figure 5A). When the percentages of abnormal SCC formations were analyzed, 49.42% of the oocytes collected from the HFD group had abnormal SCCs, and this percentage was reduced to 33.90% after PC administration (*p* < 0.05), which was comparable to controls (*p* > 0.05) (Figure 5B). The ratio of spindle length to oocyte diameter was also measured to evaluate spindle morphology (Figure 5C, left panel). Compared with the CTRL group, this ratio decreased in the HFD group (*p* < 0.01) and increased significantly after PC administration (*p* < 0.05) (Figure 5D). Similarly, the ratio of chromosome width to oocyte diameter in the HFD group was also significantly higher compared to the ratios in the CTRL group (*p* < 10^–4^), and these morphology defects were partially rescued after PC administration (Figure 5C, right panel, and Figure 5E). These data indicate that PC administration improved oocytes quality by correcting abnormal SCCs in HFD mice.

### PC Restored Mitochondrial Distribution, and Attenuated Both Oxidative Stress, and Early Apoptosis in Obese-Mice Oocytes

**FIGURE 6:**
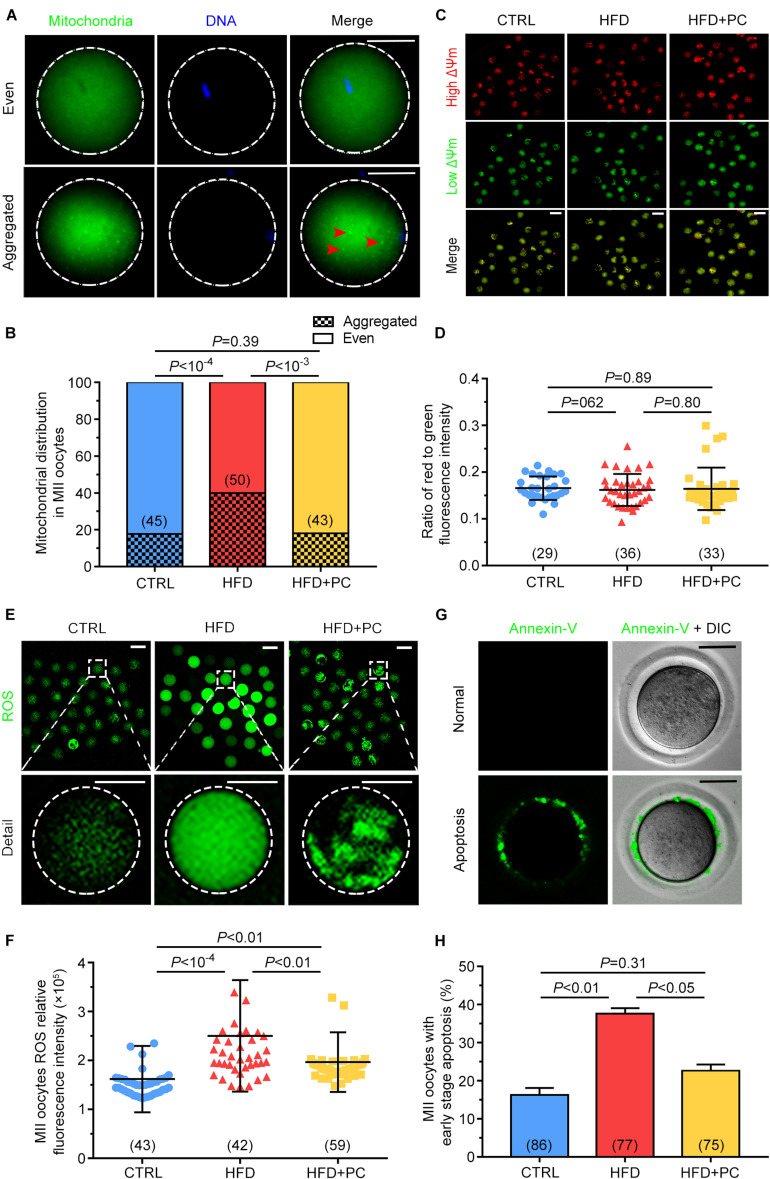
PC restored the mitochondrial distribution in oocytes, and attenuated oxidative stress and early apoptosis in obese mice. **(A)** Representative images of mitochondria with *even* or *aggregated* distributions. Red arrows indicate aggregated mitochondria. Green, mitochondria; blue, DNA. The circular white frame indicates the oocyte edge. Scale bar = 20 μm. **(B)** Percentages of MII oocytes with altered mitochondrial distributions. **(C)** Representative images of mitochondrial membrane potentials. Red, high ΔΨm; Green, low ΔΨm. Scale bar = 100 μm. **(D)** Ratio of red to green fluorescence intensities from **(C)**. **(E)** Representative images of ROS generation determined by DCFH-DA fluorescence (green). Scale bar = 100 μm. The square white frame indicates the region shown in detail, and the circular white frame indicates the oocyte edge. **(F)** Quantification of ROS fluorescence intensities. **(G)** Representative images of early stage apoptosis in MII oocytes. No green fluorescence signals were observed in oocyte membranes, indicating no apoptosis **(upper panel)**. Oocytes undergoing early apoptosis were characterized by an unambiguous green membrane signal **(lower panel)**. Scale bar = 100 μm. **(H)** Percentages of oocytes undergoing early stage apoptosis. Data are expressed as mean ± SD and were compared by one-way ANOVA and Newman–Keuls *post hoc* tests. The number of oocytes assessed in each group is shown in parentheses.

As mitochondria play a pivotal role during oocyte maturation, their distributions and membrane potentials were examined in MII oocytes. Mitochondrial distributions were classified as either *Even* or *Aggregated*, indicating either an evenly distributed pattern or an aggregated pattern in MII-stage oocytes (Figure 6A). Only 22.2% of MII oocytes in the CTRL group showed an aggregated distribution, but this proportion increased to 70.0% in the HFD group, significantly higher than in the CTRL group (*p* < 10^–4^). After the administration of PC, the aggregated distribution proportion decreased to 30.2%, comparable to that of the CTRL group (*p* > 0.05) (Figure 6B). The JC-10 fluorescent dye, used to detect the mitochondrial membrane potential (Figure 6C), showed that there was no statistical difference among the three groups (*p* > 0.05) (Figure 6D).

Oocyte oxidative stress can be indicated by ROS levels. In order to detect the effect of obesity and PC on oocyte oxidative stress, DCFH-DA fluorescence intensity was measured to evaluate ROS levels. In MII oocytes from the HFD group, fluorescence intensity was significantly higher than the intensities in both the CTRL and the HFD + PC groups, indicating that obesity led to ROS production (Figure 6E). We quantified the relative fluorescence intensities and confirmed that ROS levels were higher in the HFD group (*p* < 10^–4^). One notable exception was that ROS levels decreased significantly when PC was administered (*p* < 0.01) (Figure 6F). These results indicate that the obesity-related production of ROS in oocytes can be partially inhibited by PC administration. Higher intracellular ROS levels can lead to early apoptosis, so we performed Annexin-V staining to determine the proportion of oocytes experiencing early apoptosis. Oocytes experiencing early apoptosis were characterized by an unambiguous green membrane signal (Figure 6G). We quantified these fluorescence signals and found that 37.64% of oocytes in the HFD group were apoptotic. After PC administration, the percentage of apoptosis oocytes decreased to 22.64%, significantly lower than the HFD group proportion (*p* < 0.05) (Figure 6H).

### PC Reversed Oocyte H3K9me3 Expression in Obese Mice

**FIGURE 7:**
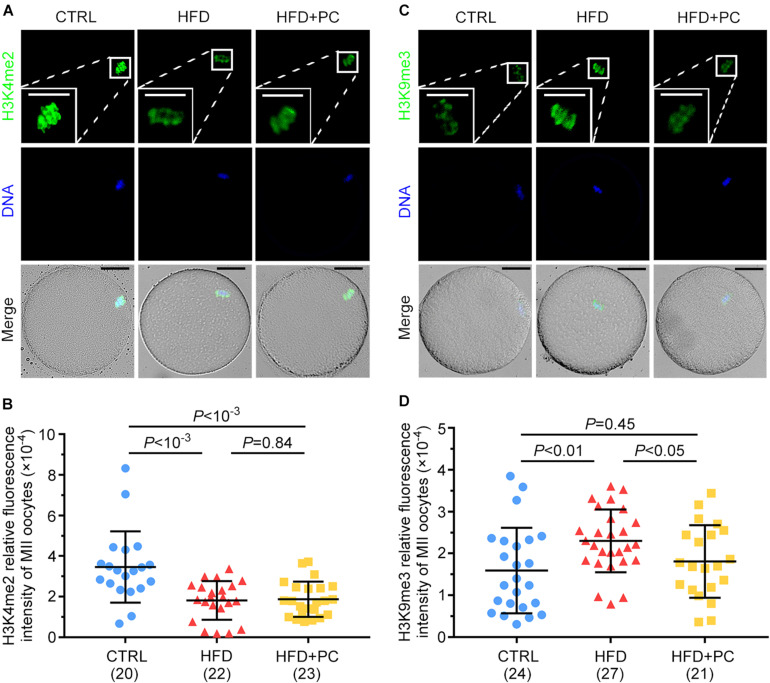
PC reversed the abnormal expression of H3K9me3 in obese mice. **(A)** Representative images of H3K4me2 inside oocytes. Green, H3K4me2; blue, DNA. The square white frame indicates the region shown in detail. Scale bar = 20 μm. **(B)** Quantified fluorescence intensities for H3K4me2. **(C)** Representative images of H3K9me3 inside oocytes. Green, H3K9me3; blue, DNA. The square white frame indicates the region shown in detail. Scale bar = 20 μm. **(D)** Quantified fluorescence intensities for H3K9me3. Data are expressed as mean ± SD and were compared by one-way ANOVA and Newman–Keuls *post hoc* tests. The number of oocytes assessed in each group is shown in parentheses.

The expression levels of H3K4me2 and H3K9me3 in MII oocytes were examined by immunofluorescence staining to assess the effect of PC on histone methylation in oocytes from HFD mice. For H3K4me2, relatively higher intensities of immunofluorescence were observed in MII oocytes from the CTRL group, and decreased intensities were observed in both the HFD and HFD + PC groups (Figure 7A). After signal quantifications, we found that the fluorescence intensities for H3K4me2 were significantly lower in the HFD and HFD + PC groups compared to CTRL group intensities (*p* < 10^–3^) (Figure 7B). Conversely, the expression of H3K9me3 was significantly higher in oocytes from the HFD group (*p* < 0.01), and this abnormal expression was completely inhibited by PC administration (Figures 7C,D). Taken together, these results indicate that PC reversed these abnormal epigenetic modifications inside oocytes in obese mice.

## Discussion

Obesity induced by a high-fat diet is harmful to the female reproductive system. In this study, we used mice that had been fed a high-fat diet as a model system for obesity, and observed that obesity-induced adverse effects on ovary antioxidant enzymes, follicular development, oocyte maturation, and meiotic spindle morphology were responsible for the reduced litter sizes and decreased survival rates. In addition, we found that the number of births and the survival rate for offspring increased in obese female mice after gavage with PC. These results showed that intragastric administration of PC reduced ovarian antioxidant enzymes, the number of atretic follicles, oocyte meiotic errors, ROS levels, and early apoptosis in obese mice.

Recent research has shown that high-calorie diets can cause hyperphagia and promote obesity. Feeding obese mice with 2-fucosyllactose and anagliptin reduced both body weight and dietary intake (Kohno et al., 2020; Lee et al., 2020). We did observe increased body weights in mice fed a high-calorie diet, and reduced food consumption after PC gavage in obese mice, but PC administration did not reduce mouse body weight, illustrating that weight loss was not an effect of PC administration. The long-term intake of a high-fat diet can lead to an imbalance in energy metabolism and an increased burden associated with the pancreatic insulin secretion, resulting in abnormal glucose metabolism and an abnormal insulin response (Ye et al., 2018). We found that PC gavage reversed this abnormal glucose metabolism and abnormal insulin response in HFD mice. Although a detailed mechanism for this is still unclear, the reduction in food consumption after PC administration may be related to the improvements in glucose metabolism and insulin response.

In our study, reduced litter sizes and offspring survival rates were observed in obese mice. These results are consistent with a recent study that reported decreased litter survival in mice fed a high-fat diet (Smoothy et al., 2019). Our previous study found that PC gavage improved litter sizes and survival rates in D-gal-induced aging mice (Li et al., 2016). Here, similar results were obtained after PC administration in obese mice, indicating that PC can rescue reproductive disorders in HFD mice, shown by increased litter sizes and offspring viability.

Research has also found that obesity can cause a significant increase in the number of atretic follicles by triggering apoptosis in follicular cells (Wang et al., 2014). Our results show that the number of atretic follicles in HFD mice increased significantly, and their number was effectively reduced after intragastric administration of PC.

As obesity led to an increase in the number of atretic follicles, we wondered if this increase was induced by the abnormal expressions of the genes *BMP4*, *GDF9*, or *LHX8* that play key roles during ovarian follicular development (Fu et al., 2017; Liu et al., 2017; Stocker et al., 2020). Our results demonstrated that there were no significant differences in their mRNA expression levels among the three mice groups, suggesting that the increased number of atretic follicles was not caused by abnormal *BMP*, *GDF9*, and *LHX8* gene expression.

Follicular development also depends on the regulation and control of the pituitary-gonadal axis. The pituitary glands of female mice fed a HFD have been shown to secrete less endogenous hypothalamic gonadotropin releasing hormone (GnRH) (Sharma et al., 2013). As a GnRH-regulated hormone, serum FSH in the present HFD female mice increased significantly, and this increase was partially blocked after PC gavage. However, obesity did not cause any significant changes in serum E2 levels, consistent with the recent study of Li et al. (2020).

Functional ovarian damage in obese females has been associated with abnormal levels of antioxidant enzymes (Lim and Luderer, 2011), and the mammalian ovary possesses antioxidant defenses, including ROS-scavenging enzymes such as CAT, GSH-Px, and SOD (Sato et al., 1992; Hsieh et al., 1997). CAT catalyzes the decomposition of H_2_O_2_ into H_2_O and O_2_ to protect cells from H_2_O_2_ toxicity (Sun et al., 2013). GSH-Px can also reduce toxic peroxides to non-toxic hydroxyl compounds, protecting cells from oxidative damage (Alexa and Jerca, 1996). SOD is the most important free-radical scavenging substance to organisms (Ozata et al., 2002; Belviranli et al., 2018), and MDA is a marker for lipid peroxidation (Belviranli et al., 2018). Consistent with our experimental results, obesity can significantly reduce the activities of GSH-Px and SOD in mice ovaries, but their activities did not change after intragastric administration of PC, indicating that the effect of PC on fertility was not due to changes in GSH-Px and SOD activities. Similarly, it has been reported that obesity can increase CAT activity in female mice (Kleniewska and Pawliczak, 2019), also consistent with our findings. Interestingly, the administration of PC in obese mice was able to reduce CAT activity, suggesting a possible reason for PC to improve the fertility of female mice by restoring CAT activity in the ovary.

Poor oocyte quality is one of the main reasons for subfertility in obese females. Characterized by cellular fragmentation and degeneration, a high proportion of poor-quality oocytes was observed in HFD mice. As a high level of ROS can induce oocyte apoptosis (Chaube et al., 2005), the ability of PC to minimize fragmentation and degeneration is probably due to its function of inhibiting ROS production. Moreover, the decrease in PB1 extrusions as well as changes in early embryo development in HFD mice could be partially reversed by PC administration, indicating its positive influence on both nuclear and cytoplasmic maturation. These data concur with previous reports (Serke et al., 2012; Nishio and Isobe, 2015; Han et al., 2018), where melatonin-induced inhibition of ROS promoted the developmental potential of early embryos in HFD mice.

Meiotic spindle assembly is essential for accurate chromosome alignment and subsequent oocyte maturation and fertilization (Sun and Schatten, 2006). Abnormalities of the SCC during meiosis can lead to aneuploidy, genetic diseases, and birth defects (Waizenegger et al., 2000; Hassold and Hunt, 2001). The integrity of the SCC in HFD mouse oocytes was disrupted and may have been responsible for the decreased maturation and embryo development potential. However, PC administration in HFD mice stabilized SCC integrity; an improvement in oocyte quality that likely increased offspring number and their survival rate.

Mitochondrial distribution in the cytoplasm plays an important role during oocyte maturation, fertilization, and early embryo development (Van Blerkom, 2011). However, obesity induces an abnormal distribution pattern for mitochondria in oocytes, and increases compensatory responses to oxidative stress, mitochondrial biogenesis, and mtDNA replication, which eventually leads to mitochondrial dysfunction (Grindler and Moley, 2013). Previous studies have reported that the distribution of mitochondria is cytoskeleton-dependent and depends on microtubule organization (Yamochi et al., 2016). Therefore, the disruption in the distribution of mitochondria is likely the result of cytoskeletal disorders in HFD mice (Islam et al., 2016). Although the mitochondrial membrane potential was not altered in HFD mice, PC administration may have improved oocyte mitochondrial distribution via its function of stabilizing SCC integrity in HFD mice.

Our previous study showed that PC improved the distribution of mitochondria by reducing oxidative stress (Li et al., 2016). Abnormal oxidative stress has a variety of detrimental effects on oocyte and embryo development (Agarwal et al., 2005; Snider and Wood, 2019). In particular, the redox state is important for proper assembly of meiotic structures during oocyte meiosis (Wang et al., 2018). Exposure of oocytes to ROS causes mitochondrial damage, prevents embryo development (Komatsu et al., 2014), and induces both oxidative stress and early apoptosis (Yen and Klionsky, 2008). Consistent with these findings, we observed elevated ROS levels in HFD mice, which resulted in oocyte fragmentation and fertilization failure. Therefore, excess ROS production may explain the poor quality of oocytes in obese mice. As an antioxidant, PC inhibited the production of ROS in HFD mice oocytes, and the decline in ROS level played a fundamental role in improving oocyte quality in HFD mice.

Histone methylation modifications play an indispensable role in regulating gene expression during embryo development. H3K9 methylation ensures appropriate gene activation via silencing of gene transcription (Becker et al., 2017). Embryonic development can be greatly improved by reducing the expression of H3K9me3 in mice oocytes (Zhu et al., 2014). Here, the elevated H3K9me3 level in MII oocytes from HFD mice may have disturbed parental genomic reprogramming, which in turn may have led to abnormal embryo development and fetus growth. Significantly, PC restored the elevated H3K9me3 levels in oocytes caused by obesity. H3K4me2 is another key regulator of early embryonic development and a marker for transcriptional activation (Dempsey and Cui, 2019), and obesity can affect the expression level of H3K4me2. However, intragastric administration of PC did not improve the level of H3K4me2 in obese mice.

**FIGURE 8:**
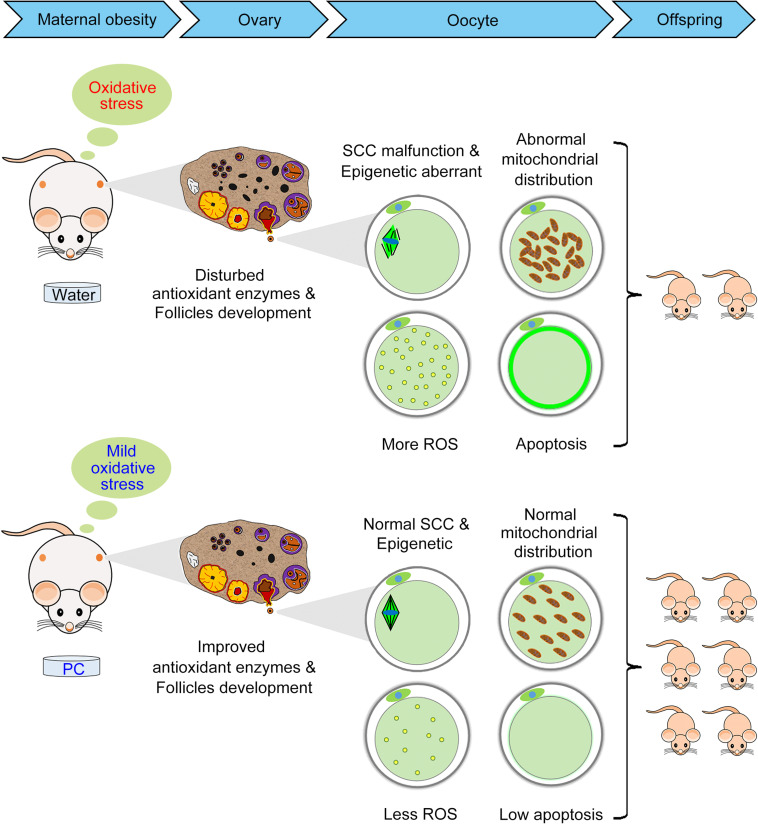
Diagram illustrating the beneficial effects of PC that improved reproductive ability in obese female mice by alleviating oxidative stress-induced ovarian and oocyte damage. The intragastric administration of PC reduced the number of atretic follicles and improved antioxidant enzymes in obese female mice ovaries. The administration of PC also rescued oocyte meiosis defects, including aberrant epigenetic effects, abnormal mitochondrial distribution, ROS accumulation, and early apoptosis. Therefore, ovary/oocyte quality, and the fertility of obese female mice was improved after PC administration.

In summary, we have shown for the first time that PC can maintain reproductive ability in obese mice induced by a HFD via its positive effects on the ovary, oocyte, and offspring births (Figure 8). Additional studies are needed to confirm the molecular mechanisms and related pathways by which PC improved ovary/oocyte quality and fertility in these obese female mice. These data have identified the beneficial effects of PC on fertility in obese females and opened a new area of research to treat human infertility.

## Data Availability Statement

The raw data supporting the conclusions of this article will be made available by the authors, without undue reservation.

## Ethics Statement

The animal study was reviewed and approved by the National Research Council Guide for the Care and Use of Laboratory Animals and were approved by the Institutional Animal Care and Use Committee at the Inner Mongolia University (Approval number: SYXK 2014-0002).

## Author Contributions

C-GL and XW: conceived and designed the experiments and analyzed the data. XW, ZH, S-JL, XH, X-JZ, X-YW, and C-JZ: performed the experiments. XW, Y-ZM, and C-GL: wrote the manuscript. All authors contributed to the article and approved the submitted version.

## Conflict of Interest

The authors declare that the research was conducted in the absence of any commercial or financial relationships that could be construed as a potential conflict of interest.
